# Maternal perceptions of the experience of attempted labor induction and medically elective inductions: analysis of survey results from listening to mothers in California

**DOI:** 10.1186/s12884-020-03137-x

**Published:** 2020-08-12

**Authors:** Eugene Declercq, Candice Belanoff, Ronald Iverson

**Affiliations:** 1 Community Health Sciences Department Boston University School of Public Health, 801 Massachusetts Ave., Boston, MA 02118 USA; 2 Department of Obstetrics and Gynecology, Boston University School of Medicine, 72 E Concord St, Boston, MA 02118 USA

**Keywords:** Labor induction, Elective induction, Cesarean section, Listening to mothers

## Abstract

**Background:**

The rate of induction of labor in the U.S. has risen from 9.6% in 1990 to 25.7% in 2018, including 31.7% of first-time births. Recent studies that have examined inductions have been small qualitative studies or relied on either medical records or administrative data. This study examines induction from the perspective of those women who experienced it, with a particular focus on the prevalence and predictors of inductions for nonmedical indications, women’s experience of pressure to induce labor and the relationship between the attempt to medically initiate labor and cesarean section.

**Methods:**

Study data are drawn from the 2119 respondents to the *Listening to Mothers in California* survey who were planning to have a vaginal birth in 2016. Mothers were asked if there had been an attempt to medically initiate labor, if it actually started labor, if they felt pressured to have the induction, if they had a cesarean and the reason for the induction. Reasons for induction were classified as either medically indicated or elective.

**Results:**

Almost half (47%) of our respondents indicated an attempt was made to medically induce their labor, and 71% of those attempts initiated labor. More than a third of the attempts (37%) were elective. Attempted induction overall was most strongly associated with giving birth at 41+ weeks (aOR 3.28; 95% C.I. 2.21–4.87). Elective inductions were more likely among multiparous mothers and in pregnancies at 39 or 40 weeks. The perception of being pressured to have labor induced was related to higher levels of education, maternal preference for less medical intervention in birth, having an obstetrician compared to a midwife and gestational ages of 41+ weeks. Cesarean birth was more likely in the case of overall induction (aOR 1.51; 95% C.I. 1.11–2.07) and especially following a failed attempt at labor induction (aOR 4.50; 95% C.I. 2.93–6.90).

**Conclusion:**

Clinicians counselling mothers concerning the need for labor induction should be aware of mothers’ perceptions about birth and engage in true shared decision making in order to avoid the maternal perception of being pressured into labor induction.

## Background

The rate of induction of labor in the U.S. has risen steadily from 9.6% in 1990 to 27.1% of all births and 37.8% of first-time births in 2018 [1, 2]. The likelihood of induction of labor varies widely by gestational age, with U.S. births at 41+ weeks for first-time mothers much more likely to involve an induction (45%) than those at 39 weeks (30%) [3]. Recently published research [4, 5] examining elective induction at 39 weeks, published after these data were collected, has suggested multiple benefits of the intervention, including lower rates of hypertensive disorders and fewer cesareans. This research, in addition to other studies which focused on the relationship between induction and cesarean section [6–10], has led to considerations of the possible wider use of elective induction to improve outcomes [11].

The multiple studies that have examined inductions in recent years have relied primarily on medical records or administrative data [12]. The preferences of women concerning induction have received less attention, with most of these studies focused on maternal experiences with post-term inductions [13]. Studies that have examined women’s experiences have been relatively small, in-depth qualitative studies [13–15], relied on survey data [16–18] or synthesized multiple qualitative studies [19–21]. A common finding has been varying levels of dissatisfaction among mothers concerning their experience of induction. Concerns expressed included a lack of information concerning the process they were about to undertake, [14, 20] frustration with the multiple delays involved in waiting for an induction [18], a sense of a loss of control in a process that they felt was done to them rather than with them [15], and a sense of being placed on someone else’s timetable [14, 21]. There were also concerns with the lack of informed, shared decision-making [19–21] and a sense of being pressured to have an induction [16]. A distinction has been made between medically indicated inductions and those not based on medical need. Einerson terms elective inductions as, “… induction in the absence of a medical or obstetric indication for delivery,“ [22] while Little adds the caveat that they occur, “…. in healthy women with a singleton pregnancy.” [23] Laughon et al. reporting one third of first time mothers and nearly half of multiparas having either elective inductions or inductions for no recorded indication [24].

This study focuses on the experiences of women who planned vaginal delivery and reported whether an induction was attempted to initiate labor. Elective inductions will be defined as those without a medical indication among mothers with a singleton birth. We report mothers’ perceptions of the experience, stratified by whether it was a medically indicated or elective induction, with particular attention to the experience of provider pressure to have an induction. It also examines the relationship between induction and cesarean birth, comparing the outcomes of attempted inductions that did or did not actually begin labor.

## Methods

The survey was developed through a collaboration of investigators from the National Partnership for Women & Families, Boston University School of Public Health and University of California, San Francisco (UCSF) Center on Social Disparities in Health, who worked with Quantum Market Research to plan and carry out the survey. The sampling frame for the *Listening to Mothers* in *California* study was drawn from California birth certificate data for births between September 1 and December 15, 2016. Women less than 18, women with out-of-hospital births, women with multiple births, non-residents of California, women who could not respond in either English or Spanish and women who were not currently living with their baby were excluded from the sample. Of the final sample, 81% participated in English and 19% in Spanish. We oversampled Black women, women with midwifery-attended births and those with vaginal births after cesarean to have sample sizes to better understand the experiences, outcomes and views of women within these smaller groups. The survey was conducted from February 22 through August 15, 2017. Participants were recruited using up to four invitation and reminder mailings and inserts incorporating elements of informed consent and cards providing information about how to participate in the survey. Those who did not respond to mailings were contacted via emails, text messages and telephone calls. Sampled women were invited to participate on their own online using any device or with an interviewer via telephone. Respondents participated from 2 to 11 months after giving birth. Of those who completed the survey, 34% did so online, 28% did so by phone with an interviewer and 39% used both methods (typically starting on their own and finishing with an interviewer) [25].

The survey questionnaire was pretested and refined in English and Spanish. On average, the survey took slightly more than 30 min to complete. The complete *Listening to Mothers in California* survey questionnaire and related materials are available at both nationalpartnership.org/LTMCA and chcf.org/listening-to-mothers-CA and the specific questions reported on here are attached as an Appendix. The core survey dataset itself is publically available at the University of North Carolina Dataverse (10.15139/S3/3KW1DB).

To better reflect a statewide profile of childbearing women aged 18 and older and giving birth to single babies in California hospitals, the final sample was weighted after the sample had been collected using demographic and other relevant variables from the 2016 Birth Statistical Master File to be representative of the full year of 2016. Our final sample size of 2539 women represented a response rate of 55%. A more detailed explanation of the methodology is presented in the *Listening to Mothers in California* full survey report appendices [25].

The Committee for the Projection of Human Subjects of California’s Office of Statewide Health Planning and Development is the Institutional Review Board (IRB) of record and approved the study and subsequent protocol amendments. The UCSF IRB also approved the project. The California Department of Public Health Vital Statistics Advisory Committee approved access to birth certificate data. We also linked to the Management Information System/Decision Support System Warehouse of the Department of Health Care Services to definitively identify women with Medi-Cal coverage, defined as Warehouse evidence of a paid claim for a 2016 birth.

In these analyses, Latina indicates women who chose “Hispanic or Latina.” “White,” “Asian and Pacific Islander” and “Black” indicate women who did not choose “Hispanic or Latina” and self-identified, respectively, as white, Asian or Native Hawaiian or other Pacific Islander, or Black. We limited the sample for this specific analysis to the 2119 women who indicated they had been planning to have a vaginal birth. We focused on attempted induction as the intervention that begins a potential cascade of other interventions rather than whether or not the induction actually began labor. We conducted bivariate analyses, examining associations between selected maternal socio-demographic characteristics, (including age, race/ethnicity, insurance, parity, body mass index (BMI), marital status, nativity, language used at home and education level), as well as maternal beliefs and preferences and attempted medical induction. Gestational age was based on the mother’s report of her due date to identify the beginning of pregnancy and the date of birth. We also examined the experience of women subsequent to receiving the attempted induction, their perception of whether they felt pressured to have an induction and the association of induction with other interventions during labor and delivery.

The determination of what was a medical induction was based on maternal responses to the question. “Why did your maternity care provider try to start your labor?” The response options were, “They were worried that I was “overdue,” “My water had broken and they worried about infection,” “The baby needed to be born soon due to a health problem (for one or both of us),” “My baby was getting too big,” “I wanted to control the timing for work or other nonmedical reasons,” “I wanted to give birth with a specific provider,” “Baby was full term: it was close to my due date,” or “Some other reason” and women could check all they felt applied. If a woman indicated one of the first three reasons it was classified as a medically indicated induction, even if she also checked one of the nonmedical indications. The open-ended responses to the “some other reason” option were also reviewed and classified as medically indicated or elective.

The multivariable analysis used logistic regression which accounted for the survey sample design (SAS v. 9.4). The first set of multivariable models examined the variables potentially related to attempted induction, elective induction and perceived pressure to have an induction. These included sociodemographic (race, age, education, parity and insurance), health (prepregnancy BMI, weeks of gestation), maternal attitude toward medical interference with labor, and a hospital-level measure of the cesarean birth rate among women with NTSV (nulliparous, term, singleton, vertex) births, divided into quartiles, as a proxy for the local maternity unit culture. A second set of models examined the relationship between medical induction, elective induction, successful and failed inductions and likelihood of cesarean section, controlling for many of the same variables.

## Results

Overall, 47% of women who had planned a vaginal birth reported their provider attempted a medical induction. Of these women, 71% reported the induction actually started labor, for a successful induction rate of 33.3%. This figure that did not differ by whether or not the induction attempt was elective (70.6 95% C.I. 65.3–75.4%) or medically indicated (72.4 95% C.I. 68.5–76.1%). The likelihood that induction started labor did not differ by maternal sociodemographic characteristics or the method used to initiate labor. Also, the likelihood of subsequent interventions (rupturing membranes after labor had begun or augmenting with Pitocin) did not vary by whether or not the attempted induction initiated labor (data not shown).

Table 1 presents the demographic distribution of both attempted inductions and, among attempted inductions, the proportions that were elective inductions. Attempt at induction was more likely among nulliparas (52%) compared to multiparas (43%), those who were obese prior to beginning pregnancy (56%) compared to those who were underweight (44%) and women attended by obstetricians (50%) compared to those attended by midwives (41%). In contrast, in the case of elective induction, multiparous mothers were more likely to report a nonmedical reason for their induction than first-time mothers (43% vs 31%). There was also a trend toward higher proportions of inductions in hospitals with higher cesarean rates, but this trend did not reach significance. There was also a steady increase in the likelihood of attempted induction across gestational ages with 71% of women at 41+ weeks reporting an attempt to medically start labor. Inductions for nonmedical reasons, however were most likely at 39 and 40 weeks (53 and 45% respectively) compared to less than 39 weeks (18%) or 41+ weeks (17%). Since the response “they were worried that my baby was overdue” was classified as a medically indicated reason for an induction, many inductions at 41+ weeks would have been coded as medically indicated.

**Table 1:** Characteristics of Women who Experienced an Attempted Medical Induction and Received an Elective Induction

Among Women who were planning to have a vaginal birth (*n* = 2119)	Any Induction Attempted (***n*** = 2119)	Elective Induction (***n*** = 970)
Demographic characteristics	**% (95% C.I.)**	**% (95% C.I.)**
**ALL RESPONDENTS**	46.8 (44.5–49.0)	37.0 (33.8–40.3)
**Race/Ethnicity**		
Latina	44.9 (41.7–48.1)	35.6 (31.0–40.4)
White	48.6 (44.-53.1)	37.2 (31.1–43.8)
Asian and Pacific Islander	48.0 (41.0–54.3)	37.1 (28.5–46.7)
Black	50.1 (42.5–57.7)	42.3 (31.9–53.4)
**Age**^a^		
< 25	44.0 (39.4–48.7)	37.2 (30.4–44.5)
25–29	45.3 (41.0–49.6)	38.1 (31.8–44.7)
30–34	49.6 (45.4–53.8)	34.8 (29.2–40.8)
35+	50.0 (44.8–55.2)	40.1 (32.9–47.6)
**Marital Status**		
Married	47.3 (44.4–50.3)	37.3 (33.1–41.8)
Living with someone	46.7 (42.3–51.1)	36.7 (30.6–43.2)
Single, never married	43.8 (37.8–50.1)	37.0 (28.2–46.7)
**Birthplace**		
US	47.5 (44.7–50.4)	38.3 (34.3–42.4)
Other country	46.0 (42.1–49.9)	33.1 (27.7–38.9)
**Education**		
High school or less	44.2 (40.2–48.3)	36.4 (30.6–42.7)
Some college	48.4 (44.4–52.5)	42.3 (36.6–48.2)
4-Year college+	48.1 (44.4–51.9)	32.8 (27.8–38.2)
**Prepregnancy BMI**^a^		
Underweight	43.9 (38.0–50.1)	39.0 (30.1–48.7)
Normal Weight	44.7 (41.1–48.3)	35.3 (30.2–40.8)
Overweight	49.0 (44.1–54.0)	40.1 (33.3–47.3)
Obese	55.8 (50.1–61.4)	34.1 (27.2–41.8)
**Insurer**		
Medi-Cal	43.5 (40.3–46.8)	36.3 (31.6–41.3)
Private insurance	49.8 (46.4–53.2)	37.3 (32.6–42.2)
**Parity**^a,b^		
1	52.0 (48.7–55.3)	31.0 (26.8–35.6)
2+	42.5 (39.5–45.6)	43.0 (38.3–47.8)
**Birth Attendant**^a^		
Obstetrician	49.5 (46.7–52.3)	39.0 (35.1–43.0)
Midwife	40.6 (35.0–46.5)	40.4 (31.7–49.8)
**Hospital NTSV CS Rate (Quartiles)**		
1 < 21.8%	44.1 (39.8–48.6)	31.6 (25.5–38.3)
2 21.9–24.2%	48.9 (44.6–53.3)	35.6 (29.6–42.0)
3 24.7–27.8%	47.0 (42.3–51.7)	38.8 (32.2–45.9)
4 > 27.9%	47.1 (42.6–51.7)	41.7 (35.3–48.5)
**Gestational Age**		
< 39 Weeks	39.1 (34.9–43.5)	18.2 (13.3–24.4)
39 Weeks	43.2 (39.2–47.3)	53.4 (47.0–59.8)
40 Weeks	51.4 (47.1–55.7)	44..5 (38.5–50.7)
41+ Weeks	70.8 (64.2–76.7)	17.3 (11.9–24.4)
**“***Birth is a process that should not be interfered with unless medically necessary”*		
Agree	46.5 (43.9–49.1)	35.6 (31.9–39.5)
Disagree or neither agree/disagree	49.1 (44.5–53.7)	41.3 (34.9–48.1)

^a^ Categories within the variable are statistically significantly different at p < .05 for likelihood of attempted induction

^b^ Categories within the variable are statistically significantly different at *p* < .05 for likelihood of elective induction

One in six (16%) women who planned to have a vaginal birth reported feeling pressure from their provider to have an induction (Table 2), with more women who actually had an induction reporting pressure (27%) than those who did not (7%). Non-Latina white women were more likely (21%) than Latina women (13%) to report feeling pressure. Non-Latina white women who had an induction were most likely to report having felt pressured (36%). Other groups reporting significantly higher levels of perceived pressure included women 35 or older (21%), those with at least a college education (21%), those who were obese prior to starting their pregnancy (23%), first time mothers (20%) and women who had reached week 41 of their pregnancy (26%). We also found almost 1 in 4 mothers (24%) who experienced an induction prior to 39 weeks reporting feeling pressure to do so, though most of those cases involved a medical indication. Among mothers with an elective induction at less than 40 weeks, 17% reported feeling pressure to do so (data not shown).

**Table 2:** Reports of Pressure to have an Induction by whether or not women had the induction Among Women who were planning to have a vaginal birth

*Did you feel pressure from any health professional to induce labor (use medicine or some other method to start your labor)?*	Felt Pressure for Induction		
	All (***n*** = 2119)	Induction Attempted (***n*** = 970)	Induction not Attempted (***n*** = 1139)
Demographic characteristics	**% (95% C.I.)**	**% (95% C.I.)**	**% (95% C.I.)**
**ALL RESPONDENTS**	16.2 (14.6–18.0)	26.5 (23.6–29.5)	6.9 (5.5–8.6)
**Race/Ethnicity**^a^			
Latina	13.0 (11.0–15.4)	21.7 (18.0–26.0)	5.0 (4.1–8.1)
White	21.2 (17.8–25.0)	36.1 (30.2–42.5)	7.2 (4.6–11.1)
Asian and Pacific Islander	15.0 (11.1–19.9)	19.5 (13.3–27.6)	9.8 (5.8–16.0)
Black	18.9 (13.7–25.5)	28.0 (19.4–38.5)	9.7 (5.1–17.9)
**Age**^a^			
< 25	11.7 (9.0–15.1)	20.0 (14.9–26.4)	5.3 (3.1–8.7)
25–29	16.1 (13.1–19.6)	28.7 (23.2–34.9)	5.5 (3.3–9.0)
30–34	16.8 (13.9–20.2)	26.2 (21.2–31.8)	6.9 (4.6–10.4)
35+	20.6 (16.8–25.1)	30.5 (24.2–37.7)	11.0 (7.3–16.2)
**Marital Status**			
Married	17.8 (15.7–20.2)	29.5 (25.7–33.7)	7.3 (5.4–9.7)
Living with someone	13.3 (10.6–16.6)	22.8 (17.9–28.6)	5.1 (3.0–8.4)
Single, never married	13.9 (10.1–18.9)	22.5 (15.3–31.6)	7.4 (4.1–13.0)
**Language**^a^			
English	19.3 (17.0–21.7)	30.6 (26.8–34.7)	8.7 (6.7–11.3)
Spanish	9.3 (6.7–13.0)	18.6 (12.9–26.2)	3.1 (1.5–6.4)
**Birthplace**^a^			
US	17.7 (15.7–20.0)	28.8 (25.2–32.6)	7.7 (5.9–10.0)
Other country	12.2 (9.9–15.0)	20.6 (16.3–25.7)	5.1 (3.2–8.1)
**Education**^a^			
High school or less	10.7 (8.5–13.5)	18.8 (14.5–24.2)	4.1 (2.4–6.8)
Some college	17.1 (14.2–20.4)	26.7 (21.8–32.2)	8.2 (5.6–11.8)
4-Year college+	20.5 (17.7–23.7)	32.3 (27.4–37.5)	9.3 (6.7–12.6)
**Prepregnancy BMI**^a^			
Underweight	17.1 (13.0–22.1)	26.9 (19.6–35.7)	8.4 (5.0–13.8)
Normal Weight	16.4 (13.9–19.3)	27.1 (22.5–32.2)	7.8 (5.5–10.8)
Overweight	11.3 (8.5–14.8)	17.5 (12.7–23.5)	5.5 (3.1–9.5)
Obese	22.8 (18.3–28.1)	34.5 (27.5–42.3)	7.3 (3.8–13.5)
**Insurer**			
Medi-Cal	14.2 (12.0–16.7)	23.3 (19.3–27.8)	7.2 (5.3–9.9)
Private insurance	18.5 (16.0–21.2)	30.0 (25.8–34.5)	6.9 (4.8–9.6)
**Parity**^a^			
1	20.1 (17.6–23.0)	30.2 (26.1–34.6)	9.0 (6.6–12.1)
2+	13.1 (11.2–15.3)	22.5 (18.8–26.7)	6.0 (4.3–8.2)
**Birth Attendant**			
Obstetrician	17.5 (15.5–19.7)	27.6 (24.2–31.3)	7.2 (5.5–9.5)
Midwife	11.6 (8.5–15.8)	20.9 (14.5–29.2)	5.3 (2.9–9.3)
**Gestational Age**			
< 39 Weeks	12.9 (10.2–16.2)	24.3 (18.8–30.8)	5.4 (3.3–8.6)
39 Weeks	12.1 (9.7–15.1)	20.9 (16.2–26.5)	5.4 (3.4–8.5)
40 Weeks	18.3 (15.2–21.9)	26.7 (21.8–32.4)	8.7 (5.8–13.0)
41+ Weeks	26.4 (20.6–33.1)	30.7 (23.4–39.0)	b
**“***Birth is a process that should not be interfered with unless medically necessary”* ^a^			
Agree	17.9 (16.0–20.0)	29.3 (25.8–33.3)	7.8 (6.1–9.9)
Disagree or neither agree/disagree	12.2 (9.6–15.4)	19.2 (14.7–24.7)	5.3 (3.0–9.1)

a Categories within the variable are statistically significantly different at p < .05

b Less than 20 cases

Among women who experienced an attempted induction, more than one-third (35%) with a pre-pregnancy Body Mass Index (BMI) of 30+ reported pressure, with 39% of non-Latina white mothers (95% C.I. 25.8–53.0%) compared to only 17% (95% C.I. 13.1–23.9%) of Latina mothers with a pre-pregnancy BMI of 30+ reporting pressure (data not shown). Women who had not given birth by 41 weeks were twice as likely (26% vs 13%) to report feeling pressure compared to women who gave birth before the 40th week of gestation. Women who thought birth shouldn’t be interfered with unless medically necessary were more likely (18% vs 12%) than those who did not express this belief to report feeling pressure.

**Fig. 1:**
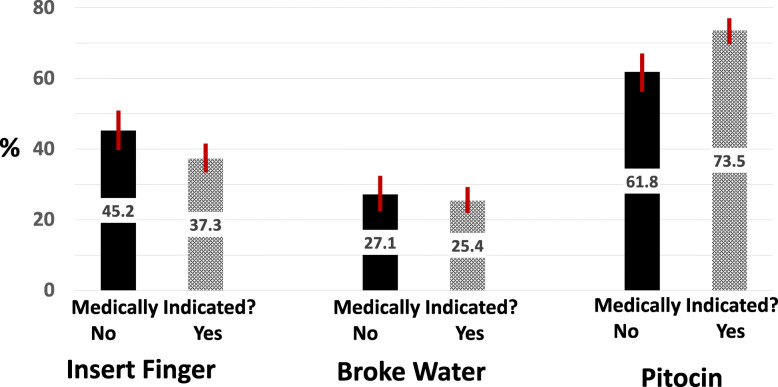
Process Used^a^ by Whether Induction was for a Medical Indication. a. Figures do not total 100% because women were asked to indicate all that were used

Women were asked about the methods of induction and given 3 choices: “Inserted a finger into the opening to my womb to ‘sweep’ or ‘strip’ the membranes loose,” “Broke and released my water before the start of labor contractions,” “Gave the medicine Pitocin through an IV drip before the start of labor contractions.” They were also able to answer “other” in cases of vaginal or oral induction agents. Women could check as many responses as applied to their situation. Among women who planned a vaginal birth and had an attempted induction, 69% reported the use of Pitocin, 40% indicated sweeping membranes and 26% breaking water. Figure 1 shows the prevalence of induction method by whether or not the induction was medically indicated. Stripping membranes was more common in cases of elective induction, though not significantly, while Pitocin was more common (74%) in medically indicated induction than in elective inductions (62%).

**Fig. 2:**
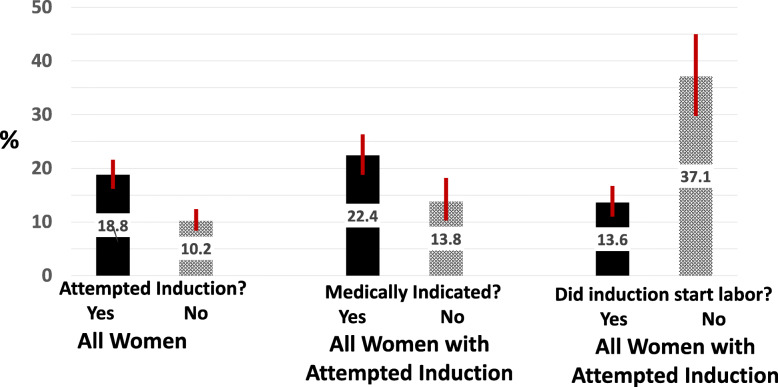
Cesarean Rate^a^ by Induction Status and Medical Indication. a. Among mothers planning a vaginal birth at full term (37+ weeks)

Among women planning a vaginal birth, 19% of those who experienced an attempted induction went on to have a cesarean while 10% of those without an attempted induction had a cesarean (Fig. 2). Cesareans were significantly more likely if the induction was for a medical indication (22%) than if it was not (14%). There was a notable difference in cesarean rates when the attempted induction successfully began labor (14%) and when it did not (37%).

In the multivariable analysis, the bivariate relationships involving induction (Table 3) were largely reaffirmed. Gestation of 41+ weeks (adjusted Odds Ratio – [aOR] 3.28; 95% C.I. 2.21–4.87), pre-pregnancy obesity, having an obstetrician rather than a midwife as birth attendant and being nulliparous all had a significant relationship to induction attempt. There were fewer strong relationships with elective induction, with births at gestational ages other than 39 weeks all less likely to involve an elective induction while first time mothers were also less likely (aOR 0.55; 95% C.I. 0.38–0.79) to have an elective induction. Overall, women reporting pressure to have an induction were more likely to have completed more years of education, have a gestational age of 41+ (aOR 2.51; 95% C.I. 1.58–3.98) or 40 weeks, report a pre-pregnancy weight that put them in the obese category or felt birth is a process that shouldn’t be interfered with unless medically necessary.

**Table 3:** Adjusted Odds Ratios ^a,b^ for Attempted Induction, Elective Induction, Pressure for Induction

Variable	Attempted Induction (*n* = 2119)	Elective Induction (*n* = 767)	Pressure to Have an Induction (*n* = 2119)
**DEMOGRAPHICS**			
*Race/Ethnicity*			
Latina (ref)			
Asian	1.11 (0.77–1.61)	1.68 (0.92–3.05)	0.74 (0.44–1.25)
White	0.89 (0.67–1.17)	1.37 (0.89–2.13)	1.18 (0.81–1.73)
Black	0.99 (0.67–1.46)	1.95 (0.96–3.96)	0.95 (0.55–1.63)
*Mother’s Education*			
HS or less	0.99 (0.71–1.37)	1.31 (0.77–2.24)	**0.38 (0.23–0.61)**
Some College	1.13(0.84–1.51)	**1.62 (1.03–2.54)**	0.73 (0.50–1.07)
College grad + (ref)			
*Mother’s Age*			
< 25	0.99 (0.72–1.37)	1.23 (0.71–2.14)	0.79 (0.49–1.28)
25–29 (ref)			
30–34	1.20 (0.90–1.60)	1.16 (0.72–1.86)	1.08 (0.73–1.61)
35+	**1.44 (1.04–1.98)**	1.15 (0.69–1.92)	**1.61 (1.06–2.45)**
*Parity*			
1	**1.40 (1.10–1.77)**	**0.51 (0.34–0.74)**	**1.42 (1.04–1.95)**
2+ (ref)			
*Body Mass Index*			
Underweight (ref)	1.13 (0.82–1.56)	1.20 (0.71–2.14)	1.19 (0.78–1.81)
Normal			
Overweight	**1.34 (1.01–1.76)**	1.27 (0.80–2.00)	0.81 (0.53–1.23)
Obese	**1.87 (1.36–2.57)**	0.97 (0.58–1.61)	**2.04 (1.34–3.12)**
**MATERNAL ATTITUDE**			
*Childbirth shouldn’t be interfered with*			
Don’t agree	1.04 (0.81–1.34)	1.40 (0.93–2.11)	**0.54 (0.37–0.80)**
Agree (ref)			
**CARE TEAM, MANAGEMENT**			
*Birth Attendant*			
Obstetrician (ref)			
Midwife	0.74 (0.54–1.01)	0.96 (0.56–1.63)	**0.54 (0.34–0.85)**
Other	0.82 (0.61–1.11)	0.73 (0.43–1.24)	1.01 (0.67–1.52)
*Gestational Age*			
< 39 weeks	0.84 (0.64–1.11)	**0.19 (0.11–0.31)**	1.15 (0.76–1.73)
39 weeks(ref)			
40 weeks	**1.35 (1.03–1.78)**	0.72 (0.48–1.10)	**1.66 (1.13–2.44)**
41+ weeks	**3.26 (2.29–4.84)**	**0.18 (0.10–0.33)**	**2.65 (1.66–4.22)**
Pregnancy Complications			
None (ref)			
At least one	1.11 (0.85–1.45)	1.58 (0.98–2.56)	1.07 (0.74–1.56)
NTSV Quartile			
21.9% (ref)			
21.9–24.2%	1.29 (0.96–1.74)	0.86 (0.53–1.41)	1.39 (0.92–2.11)
24.3–27.8%	1.14 (0.83–1.55)	1.52 (0.91–2.55)	1.04 (0.66–1.64)
27.9%+	1.25 (0.92–1.70)	**1.75 (1.04–2.94)**	**1.66 (1.09–2.54)**

a Adjusted for all other variables presented in the table.

b Comparisons that are statistically significantly different (*p* < .05) are bolded.

In examining the relationship between induction and cesarean section (Table 4), those women who had an attempted induction were 50% more likely (aOR 1.50; 95% C.I. 1.09–2.05) to have also undergone a cesarean. Among the subset of women who experienced an attempted induction, those having an elective induction were no more likely to have a cesarean, while those with an induction for a medical indication were more likely (aOR 1.75; 95% C.I. 1.23–2.48) than those women who didn’t have an attempted induction. The substantially lower cesarean rate when the attempted induction was successful compared to unsuccessful seen in the bivariate relationship was strongly confirmed in the multivariable analysis. Those women with an unsuccessful attempt at an induction were 4.4 times more likely to experience a cesarean (aOR 4.37; 95% C.I. 2.84–6.72) than mothers without an attempted induction, even after controlling for multiple demographic and health risks. Mothers reported the same rates for interventions intended to speed labor (augmentation and amniotomy) regardless of whether the induction began labor or not. The higher cesarean rate may be related to the difference in length of labor reported by mothers, which trended to being longer for those with a failed induction (mean of 18.3 h; 95% C.I. 15.4–21.1) compared to those where the induction began labor (14.7 h; 95% C.I. 13.6–15.7) (data not shown).

**Table 4:** Adjusted Odds Ratios^a,b^ for Cesarean Birth

Variable	Cesarean Birth (*n* = 2119)	Cesarean Birth (*n* = 1922)	Cesarean Birth (*n* = 1911)
**Induction Attempted**			
No (ref)			
Yes	**1.50 (1.09–2.05)**	**–**	**–**
**Elective Induction**			
Didn’t try induction (ref)			
No		**1.75 (1.23–2.48)**	
Yes	**–**	1.07 (0.68–1.68)	**–**
**Did Induction Attempt Work?**			
Did not try to induce (ref)			
Tried to induce – not sure if worked	**–**	**–**	1.60 (0.84–3.07)
Tried to induce – began labor	**–**	**–**	0.93 (0.63–1.35)
Tried to induce – labor did not begin	**–**	**–**	**4.37 (2.84–6.72)**

a Adjusted for race/ethnicity, maternal education, parity, prepregnancy BMI, gestational age, pregnancy complications, and attitude toward intervention in births

b Comparisons that are statistically significantly different (*p* < .05) are bolded.

## Discussion

We examined the experience regarding induction of labor, of 2119 women living in California who planned to have a vaginal birth in 2016 with special attention to those cases that did not involve a medical indication. We examined women’s reports of feeling pressured to have an induction and the relationship between induction and likelihood of cesarean section. Almost half (47%) of our respondents indicated an attempt was made to medically induce their labor and of those attempts, more than a third were for nonmedical reasons. The likelihood of an attempted induction was most strongly related to maternal age 35+, advanced gestational age, being a first-time mother, having an obstetrician, as compared to a midwife as a birth attendant, being nulliparous and having a pre-pregnancy BMI in the obese range. Nulliparity and gestational age either less than 39 weeks or 41+ weeks were less common among elective inductions. The reporting of feeling pressured to have an induction was related to increased gestational age, having an obstetrician as a birth attendant and pre-pregnancy obesity, but notably also related to a women’s attitude toward birth not being interfered with unless medically necessary and having at least a college degree, which may involve women’s expectations as well as the context of the perceived pressure.

Prior studies have identified an array of concerns from women undergoing induction, in particular when it was for a post term birth. In this study, women’s reports of both attempted induction and feeling pressured to have an induction increased steadily as the pregnancy passed 39 weeks. We saw a steady increase in attempted induction, with 71% of women at 41+ weeks reporting an attempted induction. While reports of pressure were greatest at 41+ weeks, mothers at 40 weeks also reported significantly higher levels of pressure to have an induction than those mothers who gave birth at 39 weeks. This study, involving births in 2016, predates more recent studies suggesting advantages of elective induction at 39 weeks [4, 5, 11, 26]. The fact that women who felt birth was a normal process and were better educated reported higher levels of pressure suggests the need for providers to focus more intently on shared decision making and exhibit greater sensitivity to the expectations of all women as they consider an induction.

More than one-third (37%) of the inductions met the criteria for an elective induction. These were most common at 39 weeks gestation, when more than half of all inductions (53%) were for nonmedical reasons. Surprisingly, 18.2% of women reported undergoing elective labor induction at less than 39 weeks, a process which is not recommended by the American Congress of Obstetrics and Gynecology [27]. There was a stronger likelihood of elective induction for multiparas (43%) than primiparas (31%) with rates quite similar to those found by Laughon and colleagues (44 and 36% respectively) in their study which included both medically elective and inductions for no recorded indication and was based on medical records [24]. The development of the measure for elective induction in this study was conservative since a case was coded as elective only when nonmedical reasons for the induction (e.g. “My baby was getting too big”) were given and no medical indication was noted. Therefore this measure may represent an undercount of the use of elective inductions. Notably, some of the reasons for the induction are patient initiated (e.g. “I wanted to give birth with my provider”), but even these may involve a complex interaction between a provider and woman prenatally. A recent study has shown how the framing of choices available to women prenatally results in both a greater likelihood of an induction and the mother feeling it was her decision [28].

One limitation of the study is the difficulty in interpreting the relationship between failed induction and increased risk of cesarean. While this was not a study primarily focused on the relationship between induction and cesarean section, we did find a higher likelihood of a cesarean after an attempted induction. Among women planning to have a vaginal birth, the cesarean rate was 19% when there was an attempted induction compared to 10% when no induction was attempted, a relationship that was maintained when we controlled for a variety of demographic, attitudinal and health risk factors (aOR =1.50). There was also a strong association between failed induction and cesarean delivery (aOR = 4.37). The sequelae of failed induction deserve more attention in future studies. This group of women may reflect that those who attempted induction but failed, were sent home and later had a cesarean section or that these women had a cesarean during the induction admission because of failure to progress, other medical reasons or patient choice.

This study has several other limitations. It is based on data from a single state, albeit a state with more than 488,000 births in 2016; but California births are demographically not comparable to the nation as a whole, with 47% of them to Latina mothers, double the proportion in the U.S. [2] We did not find differences in the rate of attempted induction or elective induction by race/ethnicity, but Latina mothers were less likely than non-Latina white mothers to perceive a pressure for an induction. We had the advantage of including variables measuring attempted induction and elective induction, though both are based on maternal recall. Past research has found mothers generally quite reliable in reporting birth events [29–31], and given the nature of the events studied here (e.g. coming into the hospital to be induced), the potential for accurate recall is greater. Our measure of elective induction combined several indications into larger categories and some may question the classification of individual items, but we drew on prior research [32] and our rates of elective induction by parity were strikingly similar to those identified in a recent records based study [24]. Since some may consider stripping of membranes as less likely to lead to subsequent interventions, we did a sensitivity analysis removing the 14% of women who indicated only stripping membranes as the type of induction and found minimal differences in results. Finally, when examining pressure for an induction we looked only at women’s perception of provider pressure and did not examine if mothers were pressuring providers to perform an induction.

This study’s data were collected prior to recent publications advocating for induction at 39 weeks [4, 11, 26] and yet almost one in four mothers (24%) who gave birth prior to 39 weeks reported feeling pressure to undergo an induction. Given the attention the recommendation concerning induction at 39 weeks received [33], it is reasonable to expect greater emphasis on inductions. Our findings would suggest the need for caution in making decisions to move to inductions too rapidly [34, 35], with an emphasis on regular two-way communication between providers and the women they serve. The tendency to “advocate while educating” is a natural one and can move patients to the viewpoint advocated by providers [28], but may have unintended consequences for parturient women and their babies. Shared decision-making tools [36] may decrease the chance that women feel unnecessary need for or pressure to undergo labor induction and result in a more positive experience and outcomes for her and her baby.

## Conclusions

Our findings suggest that a large proportion of medically induced labors are for non-medical reasons and that the attempt to clinically induce labor has a notable impact on mothers’ experiences and perceptions of their labor, including the sense of being pressured to have an induction. Clinicians counselling mothers concerning the need for labor induction should be aware of mothers’ perceptions about birth and engage in true shared decision making in order to avoid the maternal perception of being pressured into labor induction.

## Data Availability

The core dataset of the Listening to Mothers in California survey is available through the University of North Carolina Dataverse Odum Institute Data Archives (10.15139/S3/3KW1DB].

## Appendix

**Table 5:** Question Wording for Variables used in Study

Birth attendant	•Which type of maternity care provider delivered your baby on [date]? •An obstetrician-gynecologist doctor (OB or ob/gyn)
	•A family medicine doctor
	•A doctor but I’m not sure what type
	•A midwife (CNM)
	•A nurse practitioner (NP) or other nurse who is not a Midwife
	•A physician assistant (PA)
	•Other, please tell us:
Race/ethnicity	Blending of two questions:
	Which of the following best describes how you identify yourself? Please select all that apply.
	•White
	•Black or African American
	•Asian
	•American Indian or Alaskan Native
	•Native Hawaiian or Other Pacific Islander
	•Something else. Please tell us:
	Are you of Hispanic, Latina or Spanish descent?
	•Yes
	•No
Age	How old were you when your recent baby was born?
Marital status	At the time you gave birth, were you…?
	•Married
	•Living with someone as if married
	•Separated, divorced or widowed
	•Single, never married
Birthplace	In what country were you born?
	•United States
	•Some other country but born as US citizen
	•Some other country
Education	What is the highest level of education you have completed or the highest degree you have received?
	•Less than high school
	•Some high school
	•High school diploma or GED
	•Some college, but no degree
	•Associate’s degree
	•College (such as B.A., B.S.)
	•Some graduate school, but no degree
	•Graduate school (such as M.S., M.D., Ph.D.)
Insurer	At the time of your recent birth, what insurance did you have to pay for your maternity care (including provider and hospital bills)? Select all that apply.
	•Medi-Cal
	•A health plan paid for by Medi-Cal
	•Private insurance through your job or the job of your spouse, partner or parent
	•Private insurance bought from a health insurance company or plan, or through *Covered CA*
	•Other [Name of plan:]
	NOTE: In all analyses, Medi-Cal as insurer was based on a paid childbirth claim in the state Medi-Cal database
Parity	In all, how many babies have you had? Please include your new baby.
Preterm birth	Based on cross-referencing answers to questions on due date and date of delivery
Attitude Toward Birth Process	How much do you agree or disagree with the following statement? Childbirth is a process that should not be interfered with unless medically necessary. Do you…?
	•Agree strongly
	•Agree somewhat
	•Neither agree nor disagree
	•Disagree somewhat
	•Disagree strongly
Planned Vaginal	*Combination of two questions (all vaginal births plus those cases of cesareans where respondent indicated the decision was made after labor began):*
	When you recently gave birth, was your baby born vaginally or by C-section?
	•Vaginally
	•By C-section (surgery)
	You told us that your recent birth on [date] was a C-section. Was the C-section planned ahead of time, that is, was the decision to have a C-section made before you went into labor?
	•Yes, it was planned before I went into labor
	•No, the decision was made after my labor started
Any attempted labor induction	*By “inducing labor,” we mean using medicine or some other method to try to start the regular contractions of childbirth – before they start on their own.* Did your maternity care provider try to induce your labor in any way?
	•Yes
	•No
Methods of induction	How did your maternity care provider try to start your labor? Please select all that apply.
	•Inserted a finger into the opening to my womb to “sweep” or “strip” the membranes loose
	•Broke and released my water before the start of labor contractions
	•Gave the medicine Pitocin through an IV drip before the start of labor contractions
	•Tried to start my labor some other way. Please tell us:
Reasons for induction	Why did your maternity care provider try to start your labor? Please select all that apply.
	•My baby was getting too big
	•They were worried that I was “overdue”
	•My water had broken and they worried about infection
	•The baby needed to be born soon due to a health problem (for one or both of us)
	•I wanted to control the timing for work or other nonmedical reasons
	•I wanted to give birth with a specific provider
	•Baby was full term: it was close to my due date
	•Some other reason. Please tell us:
Induction start labor?	Did the medicine or other method used by your maternity care provider actually start your labor?
	•Yes
	•No
	•Not sure
Felt pressure to have an induction of labor	Did you feel pressure from any health professional to induce labor (use medicine or some other method to start your labor)?
	•Yes
	•No
Broke water (amniotomy) after labor onset^a^	During your labor, did someone break your bag of water after labor contractions had begun?
	•Yes
	•No
	•Not sure
Synthetic oxytocin to augment labor	During your labor, did someone give you the drug Pitocin (“pit” or oxytocin) to speed up labor after labor contractions had begun?
	•Yes
	•No
	•Not sure

## Abbreviations

aOR: Adjusted Odds Ratio
BMI: Body Mass Index
IRB: Institutional Review Board
